# Effectiveness of message-framing intervention on complementary feeding related behaviors among mothers with infants aged 4–8 months: a 3-arm randomized controlled trial

**DOI:** 10.1186/s13052-019-0749-0

**Published:** 2019-12-04

**Authors:** Ziba Rafieyan-Kopaei, Zohreh Fathian-Dastgerdi, Mohammad Javad Tarrahi, Fereshteh Zamani-Alavijeh

**Affiliations:** 1 Health Education and Health Promotion. Student Research Committee, School of Heath, Isfahan University of Medical Sciences, Isfahan, Iran; 2 Department of Health Education and Promotion, School of Health, Isfahan University of Medical Sciences, Hezar Jarib, Avenue, Postal Code, Isfahan, 81676-36954 Iran; 3 Department of Epidemiology and Biostatistics, School of Heath, Isfahan University of Medical Sciences, Isfahan, Iran

**Keywords:** Complementary feeding, Education of mothers, Message framing

## Abstract

**Objective:**

Complementary feeding from the age of 6 months along with breastfeeding is essential for the health of infants. The effect of educational interventions on preventing the early or late onset of complementary feeding and correcting other relevant behaviors depends on the framing of educational messages. This study aimed to compare different types of message framing on maternal behaviors in complementary feeding of infants.

**Materials and methods:**

This randomized controlled trial was performed on 96 mothers in three groups of 32 subjects with four-month-old infants in Isfahan (central Iran) during May–September 2018. Data were collected through self-report using a valid researcher-made questionnaire including demographic factors, knowledge, attitude, self-efficacy, and the complementary feeding related behaviors of mothers. For 4 months, the mothers received gain-framed messages in the GF group and loss-framed messages in the LF group from two different channels. The control group only received routine education. Data analysis was performed in SPSS20 using paired t-test, ANOVA, and Chi-square at the significance level of below 0.05.

**Results:**

In this study, all three groups were matched in terms of demographic characteristics and mean scores of **attitude**, **knowledge**, and **self-efficacy** regarding the complementary feeding of their infants. Following the intervention, the mean **knowledge** score increased in all three groups and was more favorable in the intervention groups compared to the control group. However, no difference was observed between the GF and LF groups regarding their knowledge. In addition, the GF and LF groups were similar in terms of **self-efficacy** and **behavior of mothers concerning the appropriate onset of complementary feeding**. Meanwhile, the mean scores of **attitude** and **behavior of mothers regarding the timely onset**, **diversity**, **and appropriateness of continuing complementary feeding** were higher in the LF group compared with the GF group.

**Conclusion:**

Framing messages changes the attitude and knowledge and improves behaviors related to complementary feeding. The interesting point is the different impacts of different types of framing on psychological and behavioral variables. In general, loss-framed messages have more effects on the attitude and certain parts of maternal behaviors.

**Trial registration:**

IRCT, IRCT20180901040919N1. Registered 29 October 2018, https://en.irct.ir/trial/33782

## Introduction

Complementary feeding, along with breastfeeding, is essential due to the increased food needs of infants from the age of 6 months [1]. However, complementary feeding has to be timely, adequate and appropriate as recommended by the world health organization (WHO) [2]. Hasty, delayed, or inappropriate introduction of complementary feeding endangers the health of infants. According to the literature, the early onset of complementary feeding deprives infants of the benefits of exclusive breastfeeding [3]. These infants are at risk of diseases such as diarrhea [4, 5], respiratory infections [5], allergy [6], obesity or weight gain [7] and growth impairment [1]. In addition, they become prone to chronic diseases during adulthood [4]. On the other hand, a late onset of complementary feeding cannot meet the nutritional needs of infants and leads to the emergence of malnutrition, slow growth, hindered growth, and weakness of the defense system [8].

According to WHO, the growth curve of most infants declines more along with the start of complementary feeding. According to the same report, 9.5 million deaths occurred in children under five in 2006, two-thirds of whom were in the first year of life and 35% of the mortalities were related to nutrition [1]. Evidence shows that malnutrition of children is a health problem in Iran [9, 10]. Therefore, improving the status of complementary feeding may increase the health of children and reduce the death rate of children under five by 6% [11].

Despite the mentioned outcomes, some mothers still fail to provide the necessary criteria for the complementary feeding of infants, in a way that only 39% of infants around the world are exclusively breastfed in the first 4 months of life [12]. Early onset of complementary feeding has been reported in 81.5, 78.6, and 19% of infants in Saudi Arabia, Iraq [4], and Ethiopia [3]. Therefore, education on the proper time and method of complementary feeding has to start before the age of 6 months. Early onset of complementary feeding in 80% of countries of the world, specifically the Middle East and North African countries [4] and Iran [9] involves sugar water and tea. In addition, 2.8 and 16.6% of infants in Tehran receive complementary feeding prior to the age of 4 months and from four to 6 months, respectively [13]. In Khorramabad, 51.7% of children receive complementary feeding at the age of 5 months [14]. On the other hand, a late onset of complementary feeding has been reported in 12, 21, and 39% of infants in India [15], Ethiopia [3], and certain regions of Iran (Yasuj for instance), respectively [16]. In Zabol, Iran, the mean age at onset of complementary feeding was estimated at 7.3 months [17].

According to previous studies, Ethiopian infants receive complementary feeding less than three times a day [3], and only 18.8% are fed with minimum diversity [18]. In the North West of Iran, 42.3% of children have been reported to receive the minimum nutritional diversity [19]. Some mothers fail to perform efficiently as far as the adequacy of complementary feeding is concerned. Choosing the best food to start, observing the intervals between the start of new foods and the number of times a day are also reported to affect the nutritional condition in children aged 6–12 months [20, 21]. Therefore, it is necessary to pay attention to the behavior of mothers in improving the complementary feeding of their children.

Researchers have addressed the role of certain psychological factors related to the behavior of mothers in terms of their infants’ nutrition. Some of these studies have focused on inadequate knowledge [3, 4, 21–23], while some have evaluated the role of mother’s attitudes and misconceptions [3, 9, 15, 23]. Self-efficacy, recognized as the individuals’ perception of their ability to adopt a particular behavior [24], has been reported as an important factor in behaviors associated with complementary feeding in a sample of mothers [25], hence the necessity of considering the change in such psychological factors in educational interventions in order to change the behavior of mothers and improve nutritional support.

Despite numerous educational interventions, most mothers have a low status in terms of knowledge, attitude and practice [9, 16, 19, 21] or self-efficacy in certain countries [25]. The conventional educational methods in most interventions include lectures, slideshows and films, and practical exercises that necessitate the physical presence of mothers in a place at a specific time [23, 25]. Nonetheless, some studies have shown that mothers refuse to attend in-person training programs for reasons such as the long distance to the health center [3].

Therefore, the application of new educational methods, which contrary to the traditional methods, can provide educational messages at any time and place, seems necessary to change the behavioral factors [26]. In this regard, the use of m-health has become widespread throughout the world [27, 28]. For example, it is possible to exchange educational messages in the format of photographs, films, audio files and text through Telegram, which is a widely used messaging service [29]. In such educational interventions, it is possible to formulate acceptable and easily accessible messages at desired times while spending less. Such type of education often has a better impact on the behavior and related factors compared to conventional training [30].

Creating appropriate, effective and stimulating messages is one of the main steps in educational interventions. However, the effectiveness of educational messages on motivating and changing behavior may depend on the framework of that message [31]. Different messages generate different responses [27]. Rutmann and Salvey have expressed two theories regarding frameworks, loss-framed and gain-framed messages, each of which may contribute to promoting healthy behaviors in a certain way [32]. In fact, the loss-framed framework focuses on the costs of improper behavior, whereas the gain-framed messages underline the advantages of these behaviors [33]. In the field of health, such messages have been used in many health behaviors, resulting in different outcomes. In a research by Araban for the purpose of increasing the self-efficacy of exclusive breastfeeding in mothers, both types of message frameworks significantly increased the score of mothers’ self-efficacy [28]. In another study, loss-framed messages had more effect on the maternal intent to receive MMR vaccination in comparison with gain-framed messages [34]. However, in another study, the gain-framed messages had a greater impact on mothers in terms of feeding infants with fruits and vegetables [35].

According to the above results, while the use of message frameworks may be useful for modifying or preventing a behavior, their types do not have the same impact on different behaviors. On the other hand, the role of frameworks in teaching messages has not been studied in promoting the proper behavior of mothers in child supplementation, and it is still not clear which message is to be turned into behavior in complementary feeding of infants. Therefore, this study aimed to determine and compare the effects of various types of education message frameworks of mothers on behaviors related to complementary feeding of infants.

## Methodology

### Design and sampling

This 3-arm cluster randomized controlled trial with the code of IRCT20180901040919N1 was performed to prevent the early and improper onset of complementary feeding in Isfahan (center of Iran) during May–September 2018. Subjects included mothers with infants aged 4 months to 4 months and 29 days. The infants were singleton and exclusively breastfed and had the gestational age of 37–42 weeks and no disease or disorder. In addition, there was no medical restriction to continue exclusive breastfeeding for up to 6 months of age. Other inclusion criteria were literacy, having a smartphone and Telegram, an open-source global messaging service, and Soroush, a national open-source messaging service, ability to work with these apps and consent to cooperation with the researcher. Considering a confidence interval of 95%, test power of 80%, impact size of 1.3 and standard deviation of 1.41 and 1.96, and regarding the previous research on complementary feeding [36], the sample size was estimated at 27. However, considering 15% attrition, 32 mothers were selected in each three groups. Exclusion criteria were infants with diseases, physician’s advice on the early onset of complementary feeding, having a basic problem in smartphone, leaving the Telegram and Soroush channels and lack of willingness to participate in the research. Due to the nature of the intervention, the trainer (the first author) was not blind to the allocation of groups to gain-framed and loss-framed categories. However, the participants and the statistics advisor (the third author) were blind to allocations.

### Procedure

At first, six public health centers located in downtown Isfahan, which were similar in terms of economic, social and cultural conditions, were selected and randomly divided into three groups, including GF (Gain Frame) experimental group, LF (Loss Frame) experimental group, and CG (control group). Two health centers were placed in each cluster, an assignment carried out by a lottery performed by someone outside the research team.

Afterwards, the primary list of exclusively breastfed four-month infants was prepared in each center with the help of a researcher with a bachelor’s degree in Public Health (*N* = 179). After contacting the mothers via the telephone numbers in their electronic files, 39–41 mothers remained in each group based on the inclusion criteria and were listed in the sampling framework (*N* = 121). In the next stage, 32 mothers were selected from each group (96 mothers in total) through simple random sampling and were invited to visit the health centers. After explaining the research objectives and ensuring the subjects of the confidentiality terms, a written informed consent was obtained and pre-test questionnaires were filled by mothers. A phone number was obtained from each mother, and Telegram and Soroush apps were checked by the researcher on the mobile phones of the mothers in experimental groups; the options for receiving videos, photos and text were further activated for them.

A home number was also recorded for contacting the mothers when necessary. The subjects in GF group became a member in the intervention channel with gain-framed messages, whereas mothers of the LF group joined the intervention channel with loss-gained messages. Moreover, the researcher explained how valid messages would be sent on specific days and hours with the agreement of mothers. The following approaches were employed to monitor the mothers after receiving messages in intervention groups: 1) mothers were asked to send a like to the researcher after observing and studying each message. If no likes were sent up to 3 days, the subject would be contacted by the researcher and asked for the reason; 2) multiple-choice questions were asked in the two intervention groups every 2 weeks to ensure the accurate understanding of messages and remind the content in case of a lack of proper learning; 3) all mothers were in contact with the first author (ZR-K) by asking questions and sending the picture of practices such as food preparation or drown growth chart of their children. The subjects in the control group did not join the intervention channels to receive gain-framed or loss-framed messages. However, all three groups received the in-person routine education regarding how to start and prepare several complementary foods by healthcare employees working in these centers.

### Message framing and intervention

The educational messages were designed based on the book printed by the Ministry of Health used in healthcare centers of Isfahan [37], instructions on complementary feeding by the Ministry of Health and the guidelines on complementary feeding by the world health organization [1] in two gain-framed and loss-framed forms. The messages were assessed by a panel of experts including four members of the scientific group of health education and promotion, a nutritionist, and two healthcare specialists. In addition, the ambiguities of the messages were eliminated after being studied by three mothers. Four messages were sent in a week (on Saturday, Monday, Wednesday, and Friday) based on the age of infants (from the end of 4 months to the end of 8 months) in two separate channels. The gain-and-loss messages focused on two types of outcomes: 1) the consequences of early or delayed start of complementary feeding. Before the end of 6 months, mothers in the GF group received gain-framed messages regarding the benefits of well-timed onset of complementary feeding. For instance, starting complementary feeding at the end of the 6 months, and not sooner, will better protect the health of infants against diseases. In the LF group, mothers received loss-framed messages regarding the harms of early and late onset of complementary feeding. For instance, early onset of complementary feeding increases the possibility of respiratory infections and diarrhea in infants; 2) the consequences of diversity, upsides of starting or continuing complementary feeding, and disadvantages of non-compliance. For example, salty foods have caused unhealthy eating habits in children and cardiovascular diseases and hypertension in adulthood. Text messages were sent in loss-framed and gain-framed frameworks with the background of a relevant image regarding the stages of infant feeding, how to carry out complementary feeding, stages of complementary feeding with regards to the age of infants, and how to prepare complementary food and avoid complementary feeding with harmful substances (Table 1). The framed messages were often to increase the knowledge of mothers, improve their attitude and enhance their self-efficacy and performance. Furthermore, to raise the self-efficacy of mothers, by sharing pictures of foods made by mothers in accordance with the teachings on the channel, the picture of the growth curves drawn by the mother, the experiences of mothers regarding the problems of growing or feeding the infants and how to solve those problems were used as experiences of the matched groups. At the end of each month, a pamphlet containing messages posted on the channel (a pamphlet with gain-framed and loss-framed messages) was sent to be studied by mothers and other members of the family.

**Table 1:** How to send messages to mothers

The pivotal role of messages	Type of messages	Type of distribution of messages based on the age of infants															
		Five months				Six months				Seven months				Eight months			
The importance of regular growth measurement and the use of droplets	Text framework with a fixed image	4	1					2									
Refrain from the early initiation of complementary feeding	Text framework with a fixed image		3	3	1												
The importance of breastfeeding in complementary feeding and lack of using a milk bottle and a pacifier	Text framework with a fixed image			1	3	1		1	1								
Refusal to delay the onset of complementary feeding	Text framework with a fixed image					3	1										
Stages of complementary feeding based on age	Text framework with a fixed image						1		1	1	1	1	1	1	1	1	2
How to prepare complementary food	Text framework with a fixed image						1	1	1	2	2	1	1	2	2	2	1
Avoiding complementary feeding with harmful substances	Text framework with fixed image						1		1	1	1	2	2	1	1	1	1
A demonstration of the stages of the preparation of complementary food	Seven fixed films with similar text framework						1	1		1	1	1	1				1
Summary of posted messages	Four electronic pamphlets in two frameworks				1				1				1				1
News message (complementary feeding stages based on age)	Similar text and visual framework		1			1	3		1			2				2	1

The validity of the messages was assessed in 20 mothers similar to the target group and based on the validity checklist of messages by Araban with six items scored on a Likert scale (completely agree to completely disagree). The items were: how possible it is for you to read the following messages, the following messages will motivate you, the following messages are interesting, you think that the messages below are written in a simple form, the messages are designed for you or people like you. Afterwards, the final messages were extracted. It is notable that all people had opinions of “completely agree” or “agree” for all messages [38].

### Research tools

Data collection tool was a researcher-made questionnaire designed based on questionnaires of previous studies [21, 23] and changes made based on the opinions of health education professors, a book [37] and instructions on complementary feeding by the Ministry of Health. Subsequently, the opinions of a panel of experts (eight experts in health education and promotion, two nutritionists and two personnel in comprehensive health centers), content validity index (CVI) with a score of above 0.79 [39], and content validity ratio (CVR) with scores higher than 0.56 (according to the Lavasheh Table (*N* = 12)) were used to confirm the appropriate items. In order to qualitatively provide the face validity of the tool, the items were provided for 15 people similar to the target group, and their views were analyzed by interview method. Items were corrected in terms of appropriateness and relevance of the items, the ambiguity and incorrect meanings, and the difficulty of understanding the concepts. The qualitative assessment included item scoring based on a five-point Likert scale by the same group of individuals. Following the calculation of impact score, items receiving a score above 1.5 were maintained in the questionnaire for the next analyses. To analyze the internal consistency of each part of the questionnaire, 30 mothers from the same center, who did not enter the educational intervention, answered all questions. The Brown-Spearman’s correlation between the knowledge items was 0.8, whereas the Cronbach’s alphas of attitude, self-efficacy, and behavior section were 0.87, 0.87, and 0.72, respectively.

The final questionnaire included 10 items on demographic characteristics (age and gender of infants, last delivery type of mothers, number of family members, maternal age, and maternal and paternal level of education and occupation), knowledge with scores of 0–37 containing 37 multiple-choice items (true = 1, false or no comment = 0) (for example, “which of the following foods is a better choice to start complementary feeding?”), attitude with scores of 37–185 containing 37 items scored based on a five-point Likert scale (from completely disagree = 1 score to completely agree = 5 score) (for instance, “preparing food for every meal is time consuming/futile”), and self-efficacy with the scores of 13–65 encompassing 13 items scored with the five-point Likert scale (from completely disagree = 1 to completely agree = 5) (for example, “I can prepare a food suitable to the age of my infant”).

In addition, the maternal behavior questionnaire encompassed 37 items scored based on a five-point Likert scale (from never = 1 to always = 5) with a score range of 37–185 (for instance, “I increase the frequency of complementary feeding when my child is sick”). Further measured were the total behavior scores and scores of the three subscales of observing the appropriateness of complementary feeding at the beginning with the score range of 5–25 (five items), observing the diversity and appropriateness in continuation of complementary feeding with the score range of 32–160 and 32 items, and timely onset of complementary feeding with score range of 1–5 with one item. It is notable that the questionnaires were completed through self-report, and higher scores in each scale were indicative of a better status.

However, infants growth status was also evaluated in line with the main study objectives, measuring and comparing complementary feeding behaviors and related perceptions among mothers. Birth weight and height and head circumference of children were recorded in a questionnaire, from electronic folder, at the beginning of the study (4 months old), at 6 months old, and at the end of the study (8 months old). The mothers’ performance regarding the developmental curves of children was observed at the beginning and at the end of the study by (ZR-K) and recorded in the questionnaire (with three options: none, incomplete, complete). Statistical tests were also performed to compare groups in terms of growth status and the result tables were appended as supplementary files.

### Statistical analysis

Data analysis was performed in SPSS version 20 using ANOVA (to compare the mean variables of three groups), paired t-test (to compare the mean variables prior to and following the intervention) and Chi-square (to compare the qualitative demographic variables of groups). Moreover, a *P*-value of below 0.05 was considered statistically significant.

### Ethical considerations

Ethical approvals were obtained from the Ethics Committee of Isfahan University of Medical Sciences IR.MUI.REC.1396.3.770. After explaining the research objectives, methodology, as well as the advantages and disadvantages for mothers, they were ensured of the voluntary participation in the study and the possibility of leaving the research at any desired time. In addition, the subjects were assured of the confidentiality terms regarding their personal information. A written consent was obtained from all participants, and the control group received the desired messages at the end of the educational intervention.

## Results

**Fig. 1:**
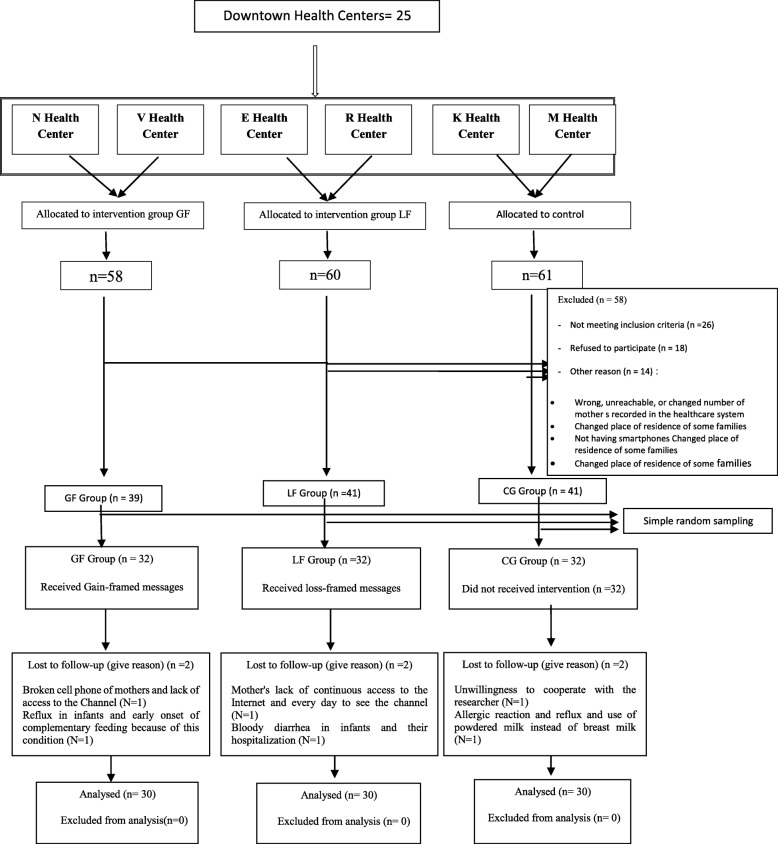
Flow diagram of the participants

In total, 96 mothers were enrolled in the study. Ultimately, 90 mothers, with a mean age of 29.87 ± 4.68 years and their infants (47 daughters and 43 sons with a mean age of 130.08 ± 7.48 days) were entered into the analysis stage (Flow Diagram in Fig. 1). The participants were homogenous in terms of demographic characteristics, including age, level of education, occupation, family size, as well as the gender and age of infants (*P* > 0.05) (Table 2). The One-way ANOVA demonstrated no significant differences between the groups prior to the intervention regarding the mean scores of **knowledge**, **attitude**, and **self-efficacy** (*P* > 0.05) (Table 3).

**Table 2:** Comparison of demographic characteristics between three groups at the beginning of the study (*N* = 90, 100%)

Variable		Total Number (%)	GF Number (%)	LF Number (%)	CG Number (%)	*P*-value
Gender	Female	47 (52.2)	19 (63.3)	12 (40)	16 (53.3)	^a^0.19
	Male	43 (47.8)	11 (36.7)	18 (60)	14 (46.7)	
Type of the last delivery of mothers	Natural	34 (37.8)	13 (43.3)	14 (46.7)	7 (23.3)	^a^0.13
	Cesarean section	56 (62.2)	17 (56.7)	16 (53.3)	23 (76.7)	
Birth rate	First	55 (61.1)	21 (70)	18 (60)	16 (53.3)	^a^0.66
	Second	31 (34.4)	8 (26.7)	10 (33.3)	13 (43.3)	
	Third	4 (4.4)	1 (3.3)	2 (6.7)	1 (3.3)	
Maternal level of education	Diploma	32 (35.6)	10 (33.3)	11 (36.7)	11 (36.7)	^a^0.99
	Associate degree	10 (11.1)	3 (10)	3 (10)	4 (13.3)	
	BSc	39 (43.3)	14 (46.7)	13 (43.3)	12 (40)	
	MSc<	9 (10)	3 (10)	3 (10)	3 (10)	
Paternal level of education	Below diploma	6 (6.7)	1 (3.3)	2 (6.7)	3 (10)	^a^0.81
	Diploma	40 (44.4)	12 (40)	15 (50)	13 (43.3)	
	Associate degree	12 (13.3)	4 (13.3)	4 (13.3)	4 (13.3)	
	BSc	26 (28.9)	9 (30)	8 (26.7)	9 (30)	
	MSc<	6 (6.7)	4 (13.3)	1 (3.3)	1 (3.3)	
Maternal occupational status	Working outside the home	20 (22.2)	7 (23.3)	7 (23.3)	6 (20)	^a^0.67
	Employed working from home	3 (3.3)	2 (6.7)	1 (3.3)	0 (0)	
	Housewife	67 (74.4)	21 (70)	22 (73.3)	24 (80)	
Paternal occupational status	Worker	14 (15.6)	4 (13.3)	4 (13.3)	6 (20)	^a^0.96
	Employee	24 (26.7)	9 (30)	8 (26.7)	7 (23.3)	
	Seller	17 (18.9)	4 (13.3)	6 (20)	7 (23.3)	
	Repairman	4 (4.4)	2 (6.7)	1 (3.3)	1 (3.3)	
	Other	31 (34.4)	11 (36.7)	11 (36.7)	9 (30)	
Growth status at four months of age	No growth impairment	79 (87.8)	29 (96.7)	26 (86.7)	24 (80)	^a^0.14
	Positive growth impairment	11(2.2)	1 (3.3)	4 (13.3)	6 (20)	
Age of infant (day) Mean ± SD		130.08 ± 7.48	130.40 ± 7.83	129.50 ± 6.89	130.33 ± 7.90	^b^0.87
Family size Mean ± SD		3.43 ± 0.58	3.33 ± 0.54	3.47 ± 0.62	3.50 ± 0.57	^b^0.50
Age of mother(year) Mean ± SD		29.87 ± 4.68	29.83 ± 4.98	29.90 ± 4.47	29.87 ± 4.74	^b^0.99

^a^Chi-square 2 (for qualitative variables)

^b^ANOVA (for quantitative variables)

**Table 3:** Comparison of behaviors and psychological variables mean scores between groups, before and after the intervention

	Variable	Groups (*N* = 30)	Time		*P*-value^*^
			Before intervention (four-month infants) Mean ± SD	After intervention (eight-month infants) Mean ± SD	
Psychological factors	Knowledge	GF	21.33 ± 5.06	30.30 ± 4.17	*p* < 0.001
		LF	20.53 ± 5.37	32.26 ± 3.27	*p* < 0.001
		Control	20.60 ± 3.38	24.80 ± 3.61	*p* < 0.001
		^a^*p*-value ^a^	*P* = 0.76	*p* < 0.001	
	Attitude	GF	139.06 ± 11.90	154.33 ± 15.14	*p* < 0.001
		LF	139.76 ± 12.65	166.76 ± 12.08	*p* < 0.001
		Control	139.13 ± 11.56	138.76 ± 14.28	0.85
		*p*-value^b^	*P* = 0.97	*p* < 0.001	
	Self-efficacy	GF	54.86 ± 8.89	61.13 ± 4.53	*p* < 0.001
		LF	55.93 ± 6.88	61.83 ± 3.27	*p* < 0.001
		Control	56.30 ± 4.89	57.56 ± 4.82	0.25
		*p*-value^b^	*P* = 0.71	*p* < 0.001	
The behavior of mothers regarding the diversity and appropriateness of onset and continuity of complementary feeding	Behavior related to initiate complementary feeding	GF	–	22.66 ± 2.83	–
		LF	–	23.16 ± 2.33	–
		Control	–	20.36 ± 3.53	–
		*p*-value^b^	–	*P* < 0.001	–
	Behavior related to diversity and appropriateness of the continuity of complementary feeding	GF	–	129.86 ± 13.12	–
		LF	–	140.10 ± 11.28	–
		Control	–	115.33 ± 9.48	–
		*p*-value^b^	–	*p* < 0.001	–
The behavior of the mother at the onset of complementary feeding	Avoid giving boiling water to infants under the age of six months	GF	–	3.97 ± 1.09	–
		LF	–	4.10 ± 1.15	–
		Control	–	2.87 ± 1.22	–
		*p*-value^b^	–	*p* < 0.001	–
	Avoid giving sugar water to infants under the age of six months	GF	–	4.90 ± 0.30	–
		LF	–	4.77 ± 0.81	–
		Control	–	4.77 ± 0.56	–
		*p*-value^b^	–	*P* = 0.61	–
	Start of complementary feeding at the end of six months	GF	–	4.33 ± 1.51	–
		LF	–	4.60 ± 1.22	–
		Control	–	3.53 ± 1.96	–
		*p*-value^b^	–	*P* = 0.03	–
Total behavior		GF	–	152.53 ± 13.71	–
		LF	–	163.26 ± 12.55	–
		Control	–	135.70 ± 9.98	–
		*p*-value ^b^	–	*p* < 0.001	–

^a^Paired sample t-test ^b^ANOVA

After the intervention, the paired t-test results demonstrated a significant increase in the mean score of **knowledge** in all three groups, compared to before the intervention (*P* < 0.001). Nevertheless, mean scores of **attitude** and **self-efficacy** significantly increased after the intervention only in the two experimental groups (*P* < 0.001). In the control group, no significant difference was observed in the mean of the mentioned variables before and after the intervention (*P* > 0.05) (Table 3). In addition, while the mean score of **attitude** increased in GF and LF groups (*P* < 0.001), it decreased in the control group, which was not significant (*P* = 0.85).

After the intervention, there was a significant difference between the three groups regarding mean scores of **knowledge**, **attitude**, **behavior related to observing the onset of complementary feeding** and **behavior related to observing the diversity and appropriateness of continuing complementary feeding** and **self-efficacy** (Table 3). Following POSTHOC (Tukey HSD), ANOVA showed a lack of significant difference between GF and LF groups concerning the mean score of **knowledge** (*P* = 0.10), **self-efficacy** (*P* = 0.80), and **behavior of mothers in observing the appropriateness of complementary feeding onset** (*P* = 0.78). However, the mean scores of each experimental group were significantly different from the scores of the control group (*P* < 0.05).

In other words, the gain-framed and loss-framed messages equally increased the scores of **knowledge**, **self-efficacy** and **behavior of mothers in observing the appropriateness of the onset of complementary feeding**. However, their impact was higher compared to routine education in the control group. According to the results, the GF, LF, and control group were significantly different in the mean scores of **mothers’ behavior regarding observing the diversity and appropriateness of continuing complementary feeding** and **attitude** (*P* < 0.001). In this regard, the loss-framed messages increased the mentioned scores more than the gain-framed messages (*P* = 0.002). However, the mean of these scores was significantly different between the GF and LF groups and the control group (*P* < 0.001) (Table 3).

Following the intervention, ANOVA and Tukey tests showed no significant difference between (GF and LF groups (*P* = 0.79)) and (GF and control groups (*P* = 0.13)) in terms of the maternal behavior score regarding the **timely onset of complementary feeding**. Nevertheless, the score of **observing the timely onset of complementary feeding at the age of six months** was significantly higher in the LF group in comparison with the control group (*P* = 0.03). In addition, no significant difference was found between GF and LF groups in terms of **using cooled down boiled water** prior to the age of six months (*P* = 0.89). However, there was a significant difference between the GF (*P* = 0.001) and LF (*P* < 0.001) groups and the control group regarding **no use of cooled down boiled water**. The mean score of **mothers’ performance in avoiding giving sugar water to infants** was higher, though not significantly (*P* > 0.05), in the two experimental groups after the intervention (Table 3).

**Fig. 2:**
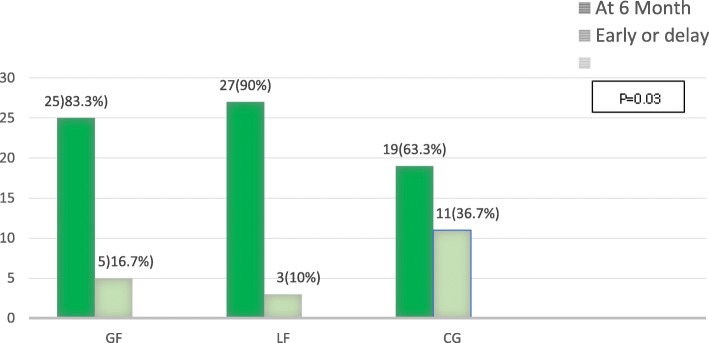
The starting time of complementary feeding

Figure 2 compares the frequency distribution of children according to the starting time of complementary feeding between the three groups. Frequency of exclusive breastfeeding until 6 months was significantly higher in the loss-framed group (P = 0.03).

The ANOVA and Tukey tests showed that mothers’ behavior towards avoiding complementary food instead of breastfeeding meals was significantly better than the control group (*P* = 0.01).

No significant difference was observed between the GF, LF, and control group of children regarding the variables of growth status and trend of weight, height and head circumference (Additional file 1: Table S1-S2). Neither was there a significant difference between the mothers of the three groups in terms of drawing and interpreting their children’s growth chart (Additional file 1: Table S3).

## Discussion

This randomized controlled trial aimed to determine the effect of different types of message frames on maternal behaviors and perceptions regarding the time and method of onset and continuation of complementary feeding. At the beginning of the research, mothers had exclusively fed four-month-old infants. For four months, the subjects in the GF and LF groups received gain-framed and loss-framed messages, respectively, through Telegram and Soroush channels.

Comparison of the results at the age of eight months demonstrated that the increased scores of **attitude**, **maternal behavior towards the diversity and appropriateness of continuing complementary feeding**, and **behavior of mothers towards the timely onset of complementary feeding** in their infants were higher in the LF group. Therefore, loss-framed messages were more effective than gain-framed messages. In a meta-analysis, following the evaluation of 96 articles, inconsistent results were obtained, showing that loss- and gain-framed messages made no significant difference in the attitude associated with various subjects [40].

On the other hand, Pakpour et al. conducted a research in the field of oral health and reported that loss-framed messages improved the behavior of students more than gain-framed messages [41]. This consistency between the groups might be owing to the almost similar cultural conditions since both studies were performed in Iran. In line with our findings, Baji et al. reported that gain-framed messages had a higher impact on foot self-care behavior of Iranian women with diabetes compared with gain-framed messages [27]. Moreover, Abhyankar, using the health belief model, observed that loss-framed messages had more influence on the intention of mothers to vaccinate their infants [34].

In the present research, while the score of **attitude** significantly increased in the GF and LF group, it was (insignificantly) reduced in the control group. However, the lack of increase in the attitude score in the control group, subjects of which received the normal care of the healthcare center, was an important point which requires more assessment. Nevertheless, this result could not be related to the difference in the effect of in-person and virtual method since in a research by Kashefi et al., where in-person education was carried out using educational tools such as whiteboard, the mean score of mothers’ attitudes towards the complementary feeding of infants was improved following the intervention [23]. The finding of the current research might be related to the dominant beliefs and common taboos of the society with regards to the control group since mothers are more concerned at the beginning of complementary feeding, and their attitude is more affected by these beliefs [9]. According to the theory of logical action, attitude depends on understanding the outcomes of behaviors and values of messages [42]. Therefore, it could be concluded that by framing the messages in the forms of loss and gain, we underscored the outcomes of behaviors, which improved attitude more in the experimental group compared with health recommendations on the routine care of the control group and common beliefs. In other words, the gain-framed messages were useful messages highlighting the adherence to behaviors related to the timely onset and proper preparation of complementary feeding, whereas the loss-framed messages emphasized the potentially harmful outcomes of behaviors associated with the early or late onset and inappropriate preparation of complementary feeding [32]. Therefore, both of these frameworks improved attitude. However, the increase in attitude score was significantly higher in the group receiving loss-framed messages, which requires more studies for further explanation.

Our findings demonstrated that the framing of messages changed the score of the behavior towards avoiding sugar water concerning four- to six-month-old infants in the three groups. However, the high score obtained in all groups is indicative of the promotion of this behavior in society. Nonetheless, both types of messages reduced the tendency to give cooled down boiling water to four- to six-month-old infants. Despite the control group, intervention groups paid to attention to avoid this behavior. In spite of the recommendation by the WHO [1] and the Ministry of Health in Iran [37], “giving **boiled water** or **sugar water** prior to the age of six months” is a prevalent improper behavior reported in some mothers in Iran and other countries [4, 9].

According to the results of the current study, gain- and loss-framed messages equally increased **knowledge**, **self-efficacy**, **and behavior of mothers towards observing the appropriateness of complementary feeding onset**, which was higher than the control group. In the research by Araban, loss-framed and gain-framed text messages had equal effects on the score of self-efficacy regarding exclusive breastfeeding [28]. Since the starting time of the complementary feeding somehow reflects the length of exclusive breastfeeding time, and according to the results of the present study and the Araban study on both gain-and-loss frame messages more than the routine educational messages in the control group improved self-efficacy and how to start food has been helpful [28]. It can be acknowledged that pointing out the positive consequences of this behavior or mentioning the disadvantages of not adopting it may play a role in its promotion. In the present study, however, the loss frame messages of exclusive breastfeeding time and proper breastfeeding were more effective during the complementary feeding period. Scott conducted a research to evaluate the effect of message framing on the knowledge of female students regarding cardiovascular diseases, observing that loss-framed and gain-framed messages equally increased knowledge [43]. On the other hand, Persky showed that following an educational intervention, compared to loss-framed messages, gain-framed messages had more impact on the behavior of mothers regarding the use of fruits and vegetables for children [35]. An interesting finding in a research by Van’t Rie was that gain-framed messages had more influence on the attitude and intention of eating healthy foods and avoiding fast food in individuals with a high self-efficacy. On the other hand, gain-framed messages had more effect on the mentioned behaviors in people with a low self-adequacy [44]. Comparing the results of the aforementioned studies with our findings, the mothers of the two experimental groups were similar in terms of self-efficacy score, but the loss-framed messages increased their attitude more than gain-framed messages. Therefore, it cannot be concluded that the effect of message framing type is always directly related to higher self-efficacy.

In a meta-analysis [40], a considerable dissociation existed between attitude and behavior since only one study showed the increasing effect of attitude as a mediator in changing the behavior. Such dissociation between attitude and behavior can also be applied to our findings because despite the higher effect of loss-framed messages on changing the attitude of mothers, the mean score of behavior in mothers regarding the onset of complementary feeding was higher in the group receiving loss-framed messages (23.16 ± 2.33) in comparison with the gain-framed group (22.66 ± 2.83), a difference which was not significant. Accordingly, the gap between attitude and a part of maternal behavior is probably associated with other factors since attitude was affected by the type of message but no significant difference was observed between the groups regarding this part of behavior. However, in accordance with the attitude change, the behaviors of mothers regarding the continuation of complementary feeding were significantly higher in the loss-framed group compared to the gain-framed group. It is also to be noted that the behavior improvement in the control group was due to the education by the personnel.

In line with certain studies, in the current research, that cell phones proved beneficial in educating health-related behaviors [27–29]. One reason is the barriers to the in-person participation in educational sessions, such as the distance to the education center. In general, the difference in the effectiveness of the type of messages might be due to the difference in the topic of the assessed behavior, duration or method of intervention and even the different effectiveness of gender and age groups. For instance, in one study, the gain-framed messages affected the self-adequacy of mothers more than fathers [35].

This study was designed in line with the main study objectives, measurement and comparison of complementary feeding behaviors and related perceptions among mothers of two intervention groups (GF and LF) and a control group (CG). In this regard, the sample size and duration of study were in line with these objectives; however, children growth status was evaluated and compared between three groups. Results of statistical test revealed no significant differences among the children. In the study of Kashfi, that sample size was more than the present study, weight gain in children in intervention group was significantly more than control group. In fact, the duration of follow up was 4 months after the intervention [23]. Researchers suggest future studies with larger sample sizes and long term follow ups to evaluate the effect of educational message framing on the growth index.

## Limitations

The short duration of follow-up was one of the limitations of this study, hence the recommendation that similar studies be performed with larger sample sizes and longer duration (up to one year of age) to assess the behavior of complementary feeding by mothers.

## Conclusion

According to the results of the present research, message framing intervention affects the change in attitude and perception and promotion of behaviors related to complementary feeding. However, an interesting point is the different impacts of various types of message framing on different psychological and behavioral variables. In general, loss-framed messages have more effect on the attitude and behavior of mothers. For instance, the comparison of pre-test and post-test scores of variables and scores of the three groups demonstrated that **knowledge**, **self-efficacy**, and **behavior of mothers regarding the appropriate preparation of complementary feeding at its onset** were affected by framed messages. Nonetheless, the type of framing had no impact in this regard. On the other hand, the **attitude** and **behavior of mothers regarding the diversity and appropriateness of continuing complementary feeding** and **onset of complementary feeding** were more affected by loss-framed messages.

## Supplementary information

**Additional file 1: Table S1.** Comparison of mean weight, high and head circumference at birth day, 4, 6 and 8 months (n = 30). **Table S2.** Comparison of position, trend and weight growthstatus of children at baseline, during and the end of the study (n = 30). **Table S3.** Mothers' ability to draw and interpret child growth charts among the three groups following the intervention

## Data Availability

Data used for this manuscript will be available upon reasonable request, meaning no personal identifying information can be shared by the corresponding author of this manuscript.

## Abbreviations

CG Group: Control Group
E-health: Electronic Health
GF Group: Group receiving Gain-Frame messages)
LF Group: Group receiving Loss-Frame messages
M-health: Mobile Health

## References

[1] Organization WH (2009). Infant and young child feeding: model chapter for textbooks for medical students and allied health professionals.

[2] Imdad A, Yakoob MY, Bhutta ZA (2011). Impact of maternal education about complementary feeding and provision of complementary foods on child growth in developing countries. BMC Public Health.

[3] Semahegn A, Tesfaye G, Bogale A (2014). Complementary feeding practice of mothers and associated factors in Hiwot Fana specialized hospital, eastern Ethiopia. Pan Afr Med J.

[4] Nasreddine L, Zeidan M, Naja F, Hwalla N (2012). Complementary feeding in the MENA region: practices and challenges. Nutr Metab Cardiovasc Dis.

[5] Khadivzadeh T, Parsai S (2004). Effect of exclusive breastfeeding and complementary feeding on infant growth and morbidity. East Mediterr Health J.

[6] Joseph CL, Ownby DR, Havstad SL, Woodcroft KJ, Wegienka G, MacKechnie H (2011). Early complementary feeding and risk of food sensitization in a birth cohort. J Allergy Clin Immunol.

[7] Wang J, Wu Y, Xiong G, Chao T, Jin Q, Liu R (2016). Introduction of complementary feeding before 4months of age increases the risk of childhood overweight or obesity: a meta-analysis of prospective cohort studies. Nutr Res.

[8] Organization W (2000). Complementary feeding " family foods for breastfed children".

[9] Amini M, Salarkia N, Eshrati B, Djazayery A (2013). Poor breastfeeding as a probable cause of childhood malnutrition: exploring mothers' and caregivers' views on breastfeeding via a qualitative study in Damavand. Iran Razavi Int J Med.

[10] Veghari G. The comparison of under-five-children’s nutrition status among ethnic groups in north of Iran, 1998–2013; results of a three stages cross-sectional study. Iran J Pediatr. 2015;25(4):1–7. 10.5812/ijp.2004 PMC4575791 26396693

[11] Patel A, Pusdekar Y, Badhoniya N, Borkar J, Agho KE, Dibley MJ (2012). Determinants of inappropriate complementary feeding practices in young children in India: secondary analysis of National Family Health Survey 2005–2006. Matern Child Nutr.

[12] Khanal V, Sauer K, Zhao Y (2013). Determinants of complementary feeding practices among Nepalese children aged 6–23 months: findings from demographic and health survey 2011. BMC Pediatr.

[13] Shidfar F, Montazer M, Azizi H, Darvishian M, Jahangiri N (2008). The relation between age of introduction of complementary feeding and physical growth of infants under 2 years of age in west of Tehran. Razi J Med Sci.

[14] Anbari K, Tajabadi Z, Baharvand P, Bazvand M, Khodadadi B. Evaluating Infant Complementary Feeding Pattern and Some Related Factors in Health Care Centers of khorramabad, West of Iran, in 2017. Epidemiology Biostatistics Public Health. 2018;15(2):1–8.

[15] Rao S, Swathi P, Unnikrishnan B, Hegde A (2011). Study of complementary feeding practices among mothers of children aged six months to two years-a study from coastal South India. Australas Med J.

[16] Mohammadhossini S, Hosseini N, Moghimi M, Fouladi M (2014). Knowledge and practice of mothers, regarding the supplementary nutrition of breast-fed infants, Yasuj, Iran. Iran J Neonatology IJN.

[17] Giovannini M, Riva E, Banderali G, Scaglioni S, Veehof S, Sala M (2004). Feeding practices of infants through the first year of life in Italy. Acta Paediatr.

[18] Kassa T, Meshesha B, Haji Y, Ebrahim J (2016). Appropriate complementary feeding practices and associated factors among mothers of children age 6–23 months in southern Ethiopia, 2015. BMC Pediatr.

[19] Kamran A, Sharifirad G, Nasiri K, Soleymanifard P, Savadpour M, Akbar HM (2017). Determinants of complementary feeding practices among children aged 6-23: a community based study. Int J Pediatr.

[20] Gessese D, Bolka H, Alemu Abajobir A, Tegabu D (2014). The practice of complementary feeding and associated factors among mothers of children 6-23 months of age in Enemay district. Northwest Ethiopia Nutr Food Sci.

[21] Shams N, Mostafavi F, Hassanzadeh A. Determinants of complementary feeding practices among mothers of 6–24 months failure to thrive children based on behavioral analysis phase of PRECEDE model, Tehran. J Education Health Promotion. 2016;5(24). https://www.ncbi.nlm.nih.gov/pmc/?term=Determinants+of+complementary+feeding+practices+among+mothers+of+6%E2%80%9324+months+failure+to+thrive+children+based+on+behavioral+analysis+phase+of+PRECEDE+model%2C+Tehran 10.4103/2277-9531.184565 PMC4960768 27500177

[22] Salarkia N, Amini M, Eslami Amirabadi M, Dadkhah M, Zowghi T, Heidari H, et al. Mothers' views and beliefs about the role of complementary feeding in children under the age of two in Damavand: a qualitative study. Arak Med University J. 2010;13(2):63–74.

[23] Kashfi SM, Jeihooni AK, Rezaianzadeh A, Karimi S (2014). The effect of mothers education program based on the precede model on the mean weight in children (6-12 months) at health centers in shiraz, Fars Province. Med J Islam Repub Iran.

[24] McCarter Spaulding DE, Deborah E (2001). Kearney MH parenting self efficacy and perception of insufficient breast milk. J Obstet Gynecol Neonatal Nurs.

[25] Mulualem D, Henry CJ, Berhanu G, Whiting SJ (2016). The effectiveness of nutrition education: applying the health belief model in child-feeding practices to use pulses for complementary feeding in southern Ethiopia. Ecol food Nutr.

[26] Lee R-G, Hsiao C-C, Chen K-C, Liu M-H (2005). An intelligent diabetes mobile care system with alert mechanism. Biomedical Engineering: Applications, Basis Communications.

[27] Baji Z, Zamanialavijeh F, Nouhjah S, Shakerinejad GH, Payaami SP (2016). comparing gain-and loss-framed message texting (sms) on foot self-care behaviors among women with type 2 diabetes. Payesh J.

[28] Merdasi F, Araban M, Saki MA (2017). The effect of message-framing on breastfeeding self-efficacy among nulliparous women in Shushtar. Iran Electronic Physician.

[29] Khademolhosseini F, Noroozi A, Tahmasebi R (2017). The effect of health belief model-based education through telegram instant messaging services on pap smear performance. Asian Pac J Cancer Prev: APJCP.

[30] Park E-J, McDaniel A, Jung M-S (2009). Computerized tailoring of health information. CIN: Comput, Inform, Nurs.

[31] Morowatisharifabad M, Tonekaboni NR (2008). Perceived self-efficacy in self-care behaviors among diabetic patients referring to Yazd Diabetes Research Center. J Birjand University Med Sci.

[32] Rothman AJ, Salovey P (1997). Shaping perceptions to motivate healthy behavior: the role of message framing. Psychol Bull.

[33] Fjeldsoe BS, Marshall AL, Miller YD (2009). Behavior change interventions delivered by mobile telephone short-message service. Am J Prev Med.

[34] Abhyankar P, O'connor DB, Lawton R (2008). The role of message framing in promoting MMR vaccination: evidence of a loss-frame advantage. Psychol Health Med.

[35] Persky Susan, Ferrer Rebecca A, Klein William M P, Goldring Megan R, Cohen Rachel W, Kistler William D, Yaremych Haley E, Bouhlal Sofia (2018). Effects of Fruit and Vegetable Feeding Messages on Mothers and Fathers: Interactions Between Emotional State and Health Message Framing. Annals of Behavioral Medicine.

[36] Khazaei T, Amoozeshi Z, Ahmadi S, Safamanesh B, Mahmoodi H (2006). The effect of education on mother's knowledge and practiceabout supplementary nutrition for children under one year. Mod Care J.

[37] Kolhdoz F, SHeikalIslam R. (2013). Nutritional needs of infants and providing supplementary food(Ministry of Health and Medical Education).

[38] Jasemzadeh M, Khafaie MA, Jaafarzadeh N, Araban M (2018). Effectiveness of a theory-based mobile phone text message intervention for improving protective behaviors of pregnant women against air pollution: a randomized controlled trial. Environ Sci Pollut Res Int.

[39] Devon HA, Block ME, Moley-Wright P, Ernst DM, Hayden SJ, Lazzara DJ (2007). A psychometric toolbox for testing validity and reliability. J Nurs Scholaarsh.

[40] Gallagher KM, Updegraff JA (2011). Health message framing effects on attitudes, intentions, and behavior: a meta-analytic review. Ann Behav Med.

[41] PakpourHajiagha A, Nourozi S, Yekaninejad MS, Mansouri A, Chaibakhsh S (2013). Effect of message framing on improving oral health behaviors in students in Qazvin. Iran J Isfahan Dental School.

[42] Goodarzi F, Araban M, Eslami AA, Zamani-Alavijeh F (2019). Development and psychometric evaluation of the diabetic Men’s dietary behaviors inventory based on the theory of reasoned action. Arch Public Health.

[43] Scott LB, Curbow B (2006). The effect of message frames and CVD risk factors on behavioral outcomes. Am J Health Behav.

[44] van’t Riet J, Werrij MQ, Nieuwkamp R, de Vries H, Ruiter RA (2013). Message frame and self-efficacy influence the persuasiveness of nutrition information in a fast-food restaurant. Food Qual Prefer.

