# Supplementary material for: Effectiveness of message-framing intervention on complementary feeding related behaviors among mothers with infants aged 4–8 months: a 3-arm randomized controlled trial
Source: Ital J Pediatr. 2019 Dec 4;45:158. doi: 10.1186/s13052-019-0749-0 (PMC6894219; doi:10.1186/s13052-019-0749-0)
Supplement: Supplementary file 1 — Additional file 1: Table S1. Comparison of mean weight, high and head circumference at birth day, 4, 6 and 8 months (n = 30). Table S2. Comparison of position, trend and weight growthstatus of children at baseline, during and the end of the study (n = 30). Table S3. Mothers' ability to draw and interpret child growth charts among the three groups following the intervention [file 13052_2019_749_MOESM1_ESM.docx]

Additional Table 1: Comparison of mean weight, high and head circumference at birth day, 4, 6 and 8 months (n = 30)

| Variables | | GF  Mean ± SD | LF  Mean ± SD | CG  Mean ± SD | P-value  Intervention |
| --- | --- | --- | --- | --- | --- |
| Weight | Birth day | 3.19 ± 0.39 | 3.10 ± 0.36 | 3.05 ± 0.34 | 0.72 |
|  | 4 months old | 6.47± 0.73 | 0.61 ± 6.59 | 6.56 ± 0.75 |  |
|  | 6 months old | 7.21 ± 0.84 | 7.53 ± 0.81 | 0.95 ± 7.48 |  |
|  | 8 months old | 8.21 ± 1.08 | 8.41 ± 0.84 | 8.32 ± 1.06 |  |
| High and | Birth day | 49.48 ± 2.89 | 50.11 ± 2.80 | 48.88 ± 2.20 | 0.58 |
|  | 4 months old | 62.03 ± 2.19 | 61.93 ± 4.40 | 62.51 ± 2.04 |  |
|  | 6 months old | 65.75 ± 2.37 | 66.85 ± 2.33 | 66.44 ± 2.35 |  |
|  | 8 months old | 69.53 ± 2.40 | 69.93 ± 2.11 | 69.86 ± 2.13 |  |
| Head circumference | Birth day | 34.48 ± 1.50 | 34.74 ± 1.43 | 34.33 ± 1.86 | 0.07 |
|  | 4 months old | 40.81 ± 1.62 | 41.88 ± 4.42 | 40.96 ± 1.44 |  |
|  | 6 months old | 42.70 ± 1.48 | 43.27 ± 1.34 | 42.57 ± 1.49 |  |
|  | 8 months old | 44.14 ± 1.30 | 44.74 ± 1.35 | 44.06 ± 1.41 |  |

Additional Table 2: Comparison of position, trend and weight growth status of children at baseline, during and the end of the study (n = 30)

| Child's age | | 4months old | | | | 6months old | | | | 8 months old | | | |
| --- | --- | --- | --- | --- | --- | --- | --- | --- | --- | --- | --- | --- | --- |
| Groups    Weight Growth  Variables | | GF | LF | CG | P | GF | LF | CG | P | GF | LF | CG | P |
|  |  | n  (%) | n  (%) | n  (%) |  | n  (%) | n  (%) | n  (%) |  | n  (%) | n  (%) | n  (%) |  |
| z-score | < -3 | 0  (0) | 0  (0) | 0  (0) | 0.54 | 0  (0) | 0  (0) | 0  (0) | 0.55 | 0  (0) | 0  (0) | 0  (0) | 0.88 |
|  | -3to-2 | 0  (0) | 1  (3.3) | 1  (3.3) |  | 0  (0) | 1  (3.3) | 0  (0) |  | 0  (0) | 1  (3.3) | 1  (3.3) |  |
|  | -2to+1 | 28  (93.3) | 29  (96.7) | 27  (90) |  | 27  (90) | 28  (93.3) | 28  (93.3) |  | 25  (83.3) | 25  (83.3) | 24  (80) |  |
|  | +1< | 2  (6.7) | 0  (0) | 2  (6.7) |  | 3  (10) | 1  (3.3) | 2  (6.7) |  | 5  (16.7) | 4  (13.3) | 5  (16.7) |  |
| The growth trend | Parallel to the median | 22  (73.3) | 18  (60) | 19  (63.3) | 0.33 | 16  (53.3) | 14  (46.7) | 16  (53.3) | 0.25 | 15  (50) | 12  (40) | 14  (46.7) | 0.79 |
|  | Uptrend | 7  (23.3) | 8  (26.7) | 5  (16.7) |  | 7  (23.3) | 10  (33.3) | 7  (23.3) |  | 12  (40) | 15  (50) | 12  (40) |  |
|  | Stagnant | 0  (0) | 0  (0) | 0  (0) |  | 0  (0) | 1  (3.3) | 4  (13.3) |  | 0  (0) | 1  (3.3) | 2  (6.7) |  |
|  | Decline | 1  (3.3) | 4  (13.3) | 6  (20) |  | 7  (23.3) | 5  (16.7) | 3  (10) |  | 3  (10) | 2  (6.7) | 2  (6.7) |  |
| Growth status | No irregularity | 29  (96.7) | 26  (86.7) | 24  (80) | 0.14 | 23  (76.7) | 24  (80) | 22  (73.3) | 0.83 | 26  (86.7) | 26  (86.7) | 24  (80) | 0.71 |
|  | Irregularity | 1  (3.3) | 4  (13.3) | 6  (20) |  | 7  (23.3) | 6  (20) | 8  (26.7) |  | 4  (13.3) | 4  (13.3) | 6  (20) |  |

Additional Table 3: Mothers' ability to draw and interpret child growth charts among the three groups following the intervention

|  | | draw growth charts | | interpret child growth charts | |
| --- | --- | --- | --- | --- | --- |
| Group |  | Before intervention Number (%) | After intervention Number (%) | Before intervention Number (%) | After intervention Number (%) |
| GF | None | (23.3) 7 | (3.3) 1 | (23.3) 7 | (6.7) 2 |
|  | incomplete | (23.3) 7 | (23.3) 7 | (43.3) 13 | (30) 9 |
|  | complete | (53.3) 16 | (73.3) 22 | (33.3) 10 | (63.3) 19 |
| LF | none | (33.3) 10 | (3.3) 1 | (33.3) 10 | (3.3) 1 |
|  | incomplete | (20) 6 | (10)3 | (43.3)13 | (30)9 |
|  | complete | (46.7)14 | (86.7)26 | (23.3) 7 | (66.7)20 |
| CG | none | (13.3) 4 | (10)3 | (23.3) 7 | (10)3 |
|  | incomplete | (36.7) 11 | (26.7) 8 | (43.3)13 | (56.7) 17 |
|  | complete | (50) 15 | (63.3) 19 | (33.3) 10 | (33.3) 10 |
| p-value | | 0.34 | 0.28 | 0.84 | 0.07 |
